# Surgery of the Gall-Bladder and Ducts

**Published:** 1897-12

**Authors:** J. McFadden Gaston

**Affiliations:** Atlanta, Ga.


ATLANTA

### Medical and Surgical Journal.

VOL. XIV. DECEMBER, 1897. No. 10.

L. B. GRANDY, M.D., MANAGING EDITOR.

M. B. HUTCHINS, M.D., BUSINESS MANAGER.

COLLABORATORS:
A. W. CALHOUN, M.D., LL.D., VIRGIL O. HARDON, M.D., FLOYD W. McRAE, M.D.,
A. W. STIRLING, M.D., JOSEPH EVE ALLEN, M.D., AUGUSTA, GA., AND
HENRY R. SLACK, PH.M., M.D., LAGRANGE, GA.

### *ORIGINAL COMMUNICATIONS.*

### SURGERY OF THE GALL-BLADDER AND DUCTS.*

BY J. McFADDEN GASTON, M.D.,
ATLANTA, GA.

A division of the bile channels connected with the liver, into the biliary tubules, the hepatic, cystic and common ducts is generally recognized by surgeons.

The first named collect the bile from the acini in the respective lobes of the liver and carry it through two channels which are included with the hepatic arteries and portal sinuses by the capsule of Glisson in the transverse fissure. These branches are united to form the hepatic duct which meets the cystic duct at an inclination varying slightly from a right angle, and they coalesce in the common bile duct, which enters by an oblique opening into the

*Abstract of paper presented to the Southern Surgical and Gynecological Association, at its meeting on November 9, 1897, in St. Louis Mo.

duodenum about two inches from the pyloric orifice of the stomach.

The gall-bladder, being a temporary reservoir for the bile, is a pear-shaped sac about three inches in length, which is attached to the under surface of the right lobe of the liver, with its globular fundus corresponding to the anterior margin of that organ, and its neck directed obliquely backward, terminating in the cystic duct. While this serves as a diverticulum of the hepatic secretion, a portion of this fluid is conveyed directly through the ductus communis into the duodenum. The passage of the chyme over the papilla at its outlet serves as a stimulus for the discharge of the bile accumulated in the gall-bladder, so that this becomes mingled with the alimentary mass in the duodenum.

It is found that the bile collects in the gall-bladder during the intervals of digestion; and its evacuation may be due in part to the mechanical pressure of the distended stomach against the sac on the under surface of the liver.

In the normal state, bile is not found in the stomach, and any regurgitation of it into that organ interferes with digestion, but when commingled with the pancreatic secretion in the duodenum a salutary influence is exerted in the assimilation of the food.

Notwithstanding the elaborate investigations upon the functions of the liver, and the rôle of the bile in the digestive processes, there is still great discrepancy in the opinions of physiologists and clinical observers as to the office of this product in the intestinal canal.

The painstaking experiments of Legg as to the province of the bile in the processes of digestion led him to the conclusion that "the only office which remains to it is that of emulsifying fats and of changing starch into sugar."

But this inference from experimentation is neutralized to a great extent by the weight of clinical experience in regard to its important agency in intestinal digestion.

The study of biliary derangements by observing clinical phenomena contributes more to a proper interpretation of the surgical pathology of the gall-bladder and ducts than the inferences from physiological experiments have done, and it is incumbent upon the investigator to duly weigh not only the effects of retention and diffusion of biliary elements, but the grave consequences of the entire loss of the bile to the physical organization.

Harley claims that "the uses of the bile cannot altogether be dispensed with, for animals with a biliary fistula invariably lose flesh, become emaciated and weak. The hair falls off, the bowels become irregular, while at the same time a great and almost constant discharge of foul-smelling gases takes place from the intestinal canal. At length, after a shorter or longer period, the animal sinks and dies."

In the eminently practical work on the liver by Dr. James Johnson, many important modifications of the system connected with the derangement of the hepatic function are delineated in graphic terms, while it is held that, "the consequences of the want of bile in the alimentary canal are truly momentous."

The able treatise of Murchison sets forth prominently the effects of disordered secretion of the liver, and the serious results upon the general health of any interruption in the supply of bile to the intestinal tract.

Bartholow, in his profound essay upon "Diseases of the Liver," insists upon the importance of bile in nutrition, and the marked influence on nutrition of the absence of bile from the process of digestion in the intestine; while he points out the manner in which this diversion of the bile interferes in the digestion of certain substances.

It is not improbable, however, that in some cases of temporary stoppage of the duct, either from concretions or hardened mucus, there may be a dislodgment of the obstruction with an evacuation of the contents of the sac. Again there may exist inflammation of the tissues of the duct without adhesion of its walls, and the swelling and turgescence may disappear so that the canal shall be restored to its normal state, and serve to convey the bile to its proper destination in the duodenum. Should these results not come about with medical treatment, it becomes necessary to evacuate the distended gall-bladder by an operation, with a view to establish either an external outlet or by catheterization of the tract from the sac into the duodenal canal.

The subsidence of the inflammation may thus restore the natural discharge, and under such favorable conditions the external opening may be closed, obviating a fistulous outlet. This fortunate result in securing against the waste of bile, relieves this process of a most serious objection as a radical operation.

It is requisite for the successful issue of any operative procedure, that a communication with the alimentary canal shall be established; and the temporary discharge of bile by an external opening, with a view to subsequent restoration of its natural channel and ultimate closure of the fistulous outlet, is not liable to the grave consequences of a permanent waste of the biliary secretion.

When the common bile duct remains impermeable an artificial connection of the gall-bladder with the duodenum has been proposed, as a feasible recourse for returning the bile to its appropriate destination in the alimentary tube. The claim of this cholecysto-duodenal communication to consideration rests chiefly upon the great importance of the bile in the digestive and assimilative processes, during the passage of the food through the upper portion of the small intestine.

The entire removal of the gall-bladder in cases of atrophy or degeneration of its tissues has been resorted to without serious interference with the biliary functions, except that the bile flows directly through the common duct into the duodenum instead of having a portion reserved in the storehouse by the wayside for future use.

There is of course a departure from the normal state in the delivery of bile continuously into the duodenal canal, instead of the physiological proceeding of retention for certain periods in the sac, and then being poured out as requisite for intestinal digestion. Yet, it is inferred that under the change of relations in the bile-producing act, the discharge adapts itself to the natural demand for it, and experiments have proven that the ductus communis becomes endowed with a retentive power by the spontaneous contraction of its outlet for certain periods in the intervals of digestion.

The continuous discharge of bile by a direct outlet from the gall-bladder into the duodenum, when the common duct is entirely occluded, certainly fulfills the ends of its production more satisfactorily than the formation of a cutaneous fistula for its escape externally, by which its influence upon the system is completely lost. At least this is the nearest approach to a normal supply of the biliary secretion, which can be provided under the circumstances, and thus relieves all the embarrassments of the situation.

My investigations in regard to the surgical relations of the gall-bladder to obstruction of the ducts, have appeared in the ATLANTA MEDICAL AND SURGICAL JOURNAL for August and October, 1884, and for September and November, 1885, in the *Southern Medical Record*, of Atlanta, for March, 1885, in the *Medical and Surgical Reporter* for September, 1885, and in the proceedings of the Medical Association of Georgia for 1886.

But my most elaborate papers have been published in *Gaillard's Medical Journal* for October, 1884, under the title of "Obstruction of the Gall-duct and its Bad Consequences, with Remedial Operation Suggested," and in the second volume of the "Reference Handbook of the Medical Sciences," with the captions of "Cholecystectomy," "Cholecystotomy" and "Duodeno-Cholecystostomy," and the recent supplement with title of "Surgery of the Gall-bladder and Ducts."

Those interested in the surgical treatment of the disorders of the gall-bladder and ducts are referred to the above publications for important data which cannot be presented in this paper, while the last named articles furnish the basis for the operative measures herein presented.

When the common duct becomes partially or entirely obstructed at its outlet, while the hepatic and cystic ducts are open, there is an accumulation of bile in the gall-bladder. This may lead to a greater or less dilatation of its walls, with inspissation of the fluid contents or a deposition of concretions. Yet there is generally a marked diminution of the secretion of bile following the arrest of its discharge into the duodenum, and eventually the liver ceases to yield any secretion, so that a stasis exists in the bile-producing process.

It is a well ascertained consequence of an occlusion of the cystic duct for any considerable period, that various abnormal collections in its cavity ensue, such as glairy mucus, pus or sero-purulent exudations. The presence of gall-stones in the ducts or intermixed with the accumulation in the sac, may complicate such cases, and the simple evacution of the contents does not ordinarily afford permanent relief.

The prime question for consideration is the practicability of adopting any measure of treatment which will restore the function of the liver and preserve the office of the gall-bladder. In case the derangement should prove to be irremediable, excision of the gall-bladder is indicated. Under such conditions the attention of the surgeon may be appropriately directed to the radical operation of cholecystectomy as first practiced by Von Langenbuck and since by other European surgeons.

The dissection of the adherent layers of the sac from the under surface of the liver does not seem to be called for in excision of the gall-bladder. In one of my canine experiments the outer wall of the sac underwent disintegration, while the adherent tissue was still firmly united to the under surface of the liver. Since the separation of this layer from the proper structure of the liver gives more trouble than any other step in the operation, it may be left attached after trimming off all the loose wall of the gall-bladder, and thus materially simplify the surgical procedure in cholecystectomy.

There is, doubtless, a tendency on the part of the advocates for different operations to undervalue the final cause in the respective cases. Excision or extirpation of the gall-bladder is not expected to supply the place of other processes for correcting its disorders, but is a *dernier ressort* in view of the inadequacy of any means for restoring the functions of this organ and for rehabilitation of the surrounding structures.

When the gall-bladder and cystic duct become obnoxious to the adjacent tissues so as to cause local disorders and general derangement of health, it is the legitimate province of surgery to get rid of them forthwith by extirpation. They no longer subserve the ends for which they are provided, and constitute an abnormality in the animal economy. Their removal is, therefore, rational and philosophic, in like manner as the separation of any other offending member from adjacent structures. This principle is generally adopted for the obliteration of cysts, and when degeneration of structure with decomposition of the contents of the sac is found in connection with occlusion of the gall-bladder, it belongs properly to the category of cysts, as it has no longer any of the capacities of a receptacle of the bile. Instead of retaining an offending member to propagate trouble to other parts, it is the dictate of common sense and sound judgment to sever its connection with the body.

This operation is not warranted in cases of temporary obstruction of the cystic duct, which admits of relief by mechanical or other means, excepting when disorganization of the structure of the sac is imminent. In permanent obliteration of the cystic duct, though the structure of the gall-bladder, up to the time of an exploratory operation, be intact, it is reasonable to infer that degeneration will ensue, so that it becomes a legitimate proceeding to excise it.

*Cholecystotomy.*—Under this designation are included such operative measure for the relief of disorders of the gall-bladder as may be undertaken without excision of its tissues, independent of puncturing or incising the sac through the walls of the abdomen, for the discharge of fluid collections.

Hepatic abscess originating in the organ, or resulting from dysentery, does not fall within the scope of this paper, yet when the biliary channels become affected from obstructions, with the presence of inspissated bile or concretions in the gall-bladder, a consequential abscess may form in the liver, that calls for consideration. Ziegler states that the irritant will first affect the walls of the ducts and their surroundings and set up inflammation there. Stones formed in the gall-bladder and in the ducts themselves are frequently discharged into the intestine through the common duct. But if one of them lodges or lingers in the duct retention of the bile ensues, and this may give rise to dilatation of the ducts and to infiltration of the liver with bile. The liver tissue may thereupon become degenerate or inflamed, while the parts around the impacted stone become also inflamed or even ulcerated. When such an inflammation of the ducts persists for a long time or leads to a persistent retention of the bile, the inflammatory changes extend to other parts of the liver. Brown or yellow granular biliary concretions appear in the interlobular tissue and within the lobules. The liver cells perish at various points, and inflammatory infiltrations appear within the lobules and terminate in abscess or fibroid induration.

Independent of this record of the pathological changes connected with biliary derangement, it has been noted by other trustworthy observers that the closure of the natural outlet of the bile brings many troubles in its train. Most frequently the progress of dilatation in the gall-bladder, with the consequent suppuration and disorganiztion, in the surrounding structures, indicates the serious nature of the disorder in these cases.

The aim of surgical interference is to arrest the march of the disorder, and to accomplish a cure.

In the absence of fistula and adhesions, when the history, progress of the disease, examination of the patient, and above all the presence of a biliary tumor, and exploratory puncture, reveal the presence of biliary calculi, Boeckel recommends cholecystotomy, but he holds that this operation is not otherwise justifiable. On the contrary, it is clear that we arrest the progress of the inflammatory disturbance in the tissues by evacuating the distended gall-bladder. Delay in undertaking the appropriate measure of relief brings disintegration of structure, which undermines the recuperative powers of the system; and this tendency to disorganization may be arrested by an early recognition of the nature of the case and a timely resort to a judicious operative measure. The earliest report on record of a radical operation for relief of the gall-bladder is that of Bobbs on June 15th, 1867, upon a lady thirty years old. The gall-bladder was emptied of its limpid fluid contents and several solid concretions, the incision in its walls being stitched and the ends cut close, while the sac was returned into the abdominal cavity. Her recovery was rapid and without an untoward symptom.

The practical outlook was not improved by the untoward results of the radical proceedings of Sims and Keen in their cases of dropsy of the gall-bladder. The condition of the ductus choledochus is not reported in the autopsy of Keen's case, but it is inferred from the post-mortem examination in the case of Sims, that obstruction of the common duct did not exist.

Encouragement has been drawn however even from these failures, for a repetition of similar operations and notices of the steps taken in other cases by various operators are reported in the January number of the *American Journal of the Medical Sciences* for 1879 by Keen, and in that of October, 1884, by Musser and Keen.

According to Musser there are four pronounced conditions, which are dangerous as sequences of biliary obstruction, and which may be looked upon as indications for operative interference. They are jaundice; a tumor in the right hypochondrium, presumably an enlarged gall-bladder, frequently recurring paroxysmal pain; and symptoms of suppuration.

The causes in general of stenosis in the bile-ducts are considered to be three: 1. Foreign bodies within the ducts (gall-stones, parasites, etc.) 2. Diseases of the walls of the ducts (*a*, congenital; *b*, adhesive inflammation or cicatrization of ulcers; *c*, parahepatitis). 3. Tumors, etc., external to the ducts.

The enlargement of the gall-bladder is generally pyriform in shape, or it forms a semi-globular or globular tumor, and is unusally movable, tender, elastic and fluctuating, but may have different qualities, and the intestine is never in front of the growth.

Acupuncture and aspiration in advance of opening the abdomen are advocated by Keen for the diagnosis of affections of the gall-bladder, but the resort to such exploratory punctures are not sustained by general experience. Yet it is well known that other prominent men concur in the propriety and expediency of this course, and Bartholow claims that this diagnosis can be reduced to an absolute certainty, while Harley insists strenuously upon its safety and efficiency. Being satisfied that this preliminary step is not requisite for the practical management of such cases, and that unforeseen risks attend the modes of exploration, while safer means of investigation may be relied upon for a correct diagnosis in this class of disorder, I cannot acquiesce in this measure for the detection of gall-stones in the sac, or for the removal of its fluid contents, in preference to an exploratory incision and examination with the finger. While recognizing the preliminary process, exploratory operations are sanctioned by Keen, and if the ascertained conditions warrant further steps, to be followed by cholecystotomy. He inculcates as a rule, that the incision should be made over the center of the tumor and parallel to the free border of the ribs. The greater facility of access to the tumor overbalances, in his estimation, any less hemorrhage from the linea alba. It should be sufficient for exploration, being made about three inches in length, and enlarged, if need be, afterward. All bleeding should be arrested by hemostatic forceps or catgut ligatures before opening the peritoneum. This being done by incision upon a directory to avoid wounding the sac, two fingers should be used, or if necessary the whole hand, to explore the condition of the various abdominal organs and learn the exact nature, attachment, etc., of the tumor; and so far as possible the character of its contents. The cystic and common ducts are to be examined to determine the presence and situations of gall-stones with their size, shape, and mobility. If any be found in the ducts, the suggestion of Handfield Jones to endeaver to push them into the duodenum should be tried. This failing, we should endeavor to push them back into the gall-bladder, as this part of the duct has already been dilated by their onward passage.

(*To be concluded.*)

